# Low Prevalence of Substandard and Falsified Antimalarial and Antibiotic Medicines in Public and Faith-Based Health Facilities of Southern Malawi

**DOI:** 10.4269/ajtmh.16-1008

**Published:** 2017-05-03

**Authors:** Felix Khuluza, Stephen Kigera, Lutz Heide

**Affiliations:** 1 Pharmacy Department, College of Medicine, University of Malawi, Blantyre, Malawi; 2 Mission for Essential Drugs and Supplies (MEDS), Nairobi, Kenya; 3 Pharmaceutical Institute, Eberhard-Karls-University Tuebingen, Tuebingen, Germany

## Abstract

Substandard and falsified antimalarial and antibiotic medicines represent a serious problem for public health, especially in low- and middle-income countries. However, information on the prevalence of poor-quality medicines is limited. In the present study, samples of six antimalarial and six antibiotic medicines were collected from 31 health facilities and drug outlets in southern Malawi. Random sampling was used in the selection of health facilities. For sample collection, an overt approach was used in licensed facilities, and a mystery shopper approach in nonlicensed outlets. One hundred and fifty-five samples were analyzed by visual and physical examination and by rapid prescreening tests, that is, disintegration testing and thin-layer chromatography using the GPHF-Minilab. Fifty-six of the samples were analyzed according to pharmacopeial monographs in a World Health Organization-prequalified quality control laboratory. Seven out-of-specification medicines were identified. One sample was classified as falsified, lacking the declared active ingredients, and containing other active ingredients instead. Three samples were classified as substandard with extreme deviations from the pharmacopeial standards, and three further samples as substandard with nonextreme deviations. Of the substandard medicines, three failed in dissolution testing, two in the assay for the content of the active pharmaceutical ingredient, and one failed in both dissolution testing and assay. Six of the seven out-of-specification medicines were from private facilities. Only one out-of-specification medicine was found within the samples from public and faith-based health facilities. Although the observed presence of substandard and falsified medicines in Malawi requires action, their low prevalence in public and faith-based health facilities is encouraging.

## Introduction

Access to “safe, effective, quality, and affordable essential medicines” has been included in the Sustainable Development Goals of the United Nations as Goal No. 3.8.^1^ However, the occurrence of low-quality medicines continues to be a pervasive and poorly understood problem, especially in low- and middle-income countries.^2^ The spread of falsified medicines has been addressed as a “global pandemic,”^3^ and alarming reports have been published on the possible scale and effects of this problem. A meta-analysis of 21 surveys in sub-Saharan Africa reported that 35% of antimalarial medicines failed chemical analysis, and 20% were classified as falsified.^4^ For sub-Saharan Africa alone, it was estimated that more than 120,000 deaths of under-five children annually may be associated with the consumption of poor-quality antimalarials.^5^ Yet, the recent report of the Lancet Commission on Essential Medicines recognized that “the true extent of the problem remains unknown.”^2^ As stated in a comprehensive review on substandard and falsified antimicrobial medicines,^6^ journalism remains the main source of information on this problem, with only limited available information in the scientific literature. Likewise, Heymann and others^7^ recently emphasized that “there is a dearth of high-quality, comprehensive data for the prevalence of substandard and falsified medicines.” A systematic review^8^ identified 179 full text articles on substandard and falsified medicines, yet only 44 of them measured the prevalence of such medicines (rather than presenting case reports, etc.), only 15 of these 44 articles met at least half of the quality assessment criteria defined in that systematic review, and just four of these studies used random sampling of the collection sites.

The lack of reliable data is further aggravated by the lack of a clear, internationally accepted terminology of low-quality medicines.^9^ The World Health Organization (WHO) has discontinued the use of the term “counterfeit medicines”^9^,^10^ as this legal term specifically denotes an infringement of a registered trademark but does not consider pharmaceutical quality. Until a new definition is agreed, WHO officially uses the cumbersome term “substandard/spurious/falsely labelled/falsified/counterfeit (SSFFC) medical products.”^10^,^11^ On the basis of an earlier suggestion,^9^ Nayyar and others^3^ classified poor-quality medicines into 1) falsified medicines, resulting from intentionally fraudulent manufacturing; 2) substandard medicines, resulting from unintentional errors in manufacturing; and 3) degraded medicines, which became of poor quality only after manufacturing, for example, due to inappropriate transport or storage conditions. This classification is pragmatic since different interventions are required to counteract the occurrence of these three classes of poor-quality medicines. However, when in a survey a medicine is identified which does not meet the relevant quality specifications, it may not always be possible to unequivocally assign it to one of these three classes. In the present paper, we use the term “falsified” for medicines, which contain either no or incorrect active ingredients,^12^,^13^ and “substandard” for medicines, which do not meet the specification of the relevant pharmacopeia without falling into our definition of “falsified.” This allows an unequivocal assignment of each sample based on analytical results alone, without requiring knowledge of the (honest or fraudulent) intentions of the manufacturer. However, these simplified definitions may misclassify some medicines in comparison to the current WHO definition of “falsified” medical products.^14^

Gaps in the current knowledge on the prevalence of poor-quality medicines also result from strong regional disparities in the availability of data on this problem. Most surveys on substandard and falsified medicines have been conducted in southeast Asia and in west and east Africa. However, as stated by Tabernero and others,^15^ more investigation needs to be done in the central and southern African regions as there are very few reports from these areas. This also applies to Malawi where the present study was conducted.

Although news media have suggested a massive occurrence of “counterfeit” medicines in Malawi,^16^ only a single study on this problem existed in the scientific literature before the commencement of the present investigation. That study^17^ investigated 11 samples each of paracetamol and co-trimoxazole tablets using pharmacopeial methods. It identified no falsified medicine but several substandard medicines, for example, with incorrect amounts of the active ingredient, or with insufficient dissolution of the active ingredient.

The scarcity of scientific information on the prevalence of substandard and falsified medicines in Malawi prompted us to initiate the present investigation. The objectives of this study were 2-fold: first, to generate qualitative and quantitative date on the occurrence of substandard and falsified antimalarial and antibiotic medicines in southern Malawi, with an emphasis on public health centers, which provide the major part of health care to Malawi's mostly rural population.^18^ And second, to contribute to capacity building in medicine quality analysis in Malawi; therefore, intentionally all analytical work was carried out in sub-Saharan Africa (Malawi and Kenya), a low-budget approach was used, and the execution of this study was combined with the training of national personnel.

The present study focused on antimalarial and antibiotic medicines for three reasons: first, for their public health importance in combatting life-threatening diseases; second, due to the potential contribution of poor-quality anti-infective medicines to the development of resistant pathogens^19^; and third, since most previous studies in the literature have focused on these therapeutic categories,^6^,^8^,^12^,^13^,^20^ allowing a comparison of the results of our study with those from other low- and middle-income countries.

In Malawi, approximately 60% of all health services are provided by the public health facilities, organized in three levels, that is, health centers, district hospitals, and central hospitals.^18^ Thirty-seven percent of health services are provided by facilities of the Christian Health Association of Malawi (CHAM),^18^ and only a small part by private for-profit health practitioners. Essential health care, including essential medicines, is provided free of charge in the public health facilities. In contrast, CHAM and private for-profit facilities charge for services and medicines.^18^

In the present survey, 155 medicine samples, representing six antimalarial and six antibiotic medicines, were collected from 31 different public, CHAM and private facilities in four districts of southern Malawi. Medicine quality was tested similar to the three-level approach used by the U.S. Pharmacopeial Convention in quality monitoring programs in Africa, South America, and southeast Asia,^21^ and to the methodology used by WHO in a study on medicine quality in six countries of sub-Saharan Africa.^20^ In 2016, WHO published guidelines on the conduct of surveys of the quality of medicines.^22^ Although these were not yet available when the present study was designed, the methodology used in our study is in good agreement with them. These guidelines also include a recommended “outline of the content of a survey report,” updating a previous recommendation.^23^ The structure of the present publication, and the subheadings used, follow the recommendation in these WHO guidelines.

The methodology of this study was pilot tested using samples of the antimalarial medicine sulfadoxine–pyrimethamine. That pilot study resulted in the identification of one falsified antimalarial medicine, which presented a serious risk to public health, and this finding was published in a preliminary communication.^24^ We here report the complete results of the analysis of the 155 samples investigated in this study. Notably, nearly all samples collected in the government and in the church health facilities were found to comply with the quality specifications. The results of our study may help to restore the trust of the population and the health workers in the medicines used in these facilities, and to focus the efforts of drug regulatory authorities and health policy makers on specific problem areas.

## Materials and Methods

### Survey period.

Medicine samples were collected between December 2014 and May 2015 and subsequently analyzed.

### Selection of medicines for sampling and testing.

The six antimalarial and six antibiotic medicines included in this study, as well as the preferred strength of the collected samples, are shown in Table 1. If the indicated strength was not available at a given collection site, an alternative strength was sampled, resulting in the collection of several samples of ciprofloxacin 500 mg tablets, amoxicillin/clavulanic acid 250/125 mg tablets, and cefuroxime (as axetil) 500 mg tablets. If medicines with generic and with brand names were available at a collection site, the brand name medicine was sampled. If several brand name medicines of identical composition were available, the most expensive brand name medicine was collected. In most collection sites, however, only a single type of the respective medicine was available. The preferred sampling of brand name medicines may represent a bias in favor of sampling better-quality medicines. However, in the present study, generic medicines turned out to show less quality problems than brand name medicines (see the Results section).

### Selection of study areas.

Malawi is divided into three administrative regions, that is, the northern, central, and southern region; the southern region comprises 13 districts.^25^ Of these 13 districts, four were randomly selected (Blantyre, Chikwawa, Nsanje, and Phalombe). All randomizations in this study were carried out using the RAND function of Excel (Microsoft Corp., Redmond, WA).

### Sampling design and selection of sample collection sites.

Both simple and stratified random sampling was used in the selection of the public health facilities included into this study. A complete list of public health centers was obtained from each of the four abovementioned districts (total = 59 health centers). For each of the three rural districts (Chikwawa, Nsanje, and Phalombe), two health centers were selected randomly. The fourth district, Blantyre, contains one of Malawi's urban centers. Since situations in urban and rural areas often differ,^22^ the public health centers in this district were stratified into urban and rural facilities, and two health centers were randomly selected from each stratum. Medicines were collected from the selected public health centers as well as from the public district hospitals of the respective districts. Phalombe and Blantyre districts do not have a district hospital, and samples were collected from the District Health Office instead. Blantyre district contains also a public central hospital, and medicines were also collected from there.

The staff of the selected public health facilities was asked whether CHAM health facilities, licensed pharmacies, licensed drug stores, or nonlicensed (= illegal) street vendors were located nearby the selected public facility. If this was the case, medicines were also collected from these. In Malawi, the number of CHAM facilities is lower than that of government health facilities.^26^ Eight CHAM facilities, located nearby the selected public health facilities, were included into this study. The number of licensed pharmacies in Malawi is very small (reported to be 78 pharmacies in the year 2010),^27^ and all of them are located in the urban centers. Therefore, only in Blantyre District, licensed pharmacies could be included into the present study. In each of the three rural districts, a so-called drug store was included instead. Drug stores are licensed private outlets, which do not require a pharmacist on their premises but a pharmacy technician, nurse, or clinical officer. They are not allowed to dispense prescription drugs or pharmacist-only medicines but only a limited number of common medicines.

Only few antimalarial and antibiotic medicines could be obtained from nonlicensed street vendors nearby the selected government health facilities. Unofficial enquiries for the location of such vendors were made by a locally experienced Malawian staff member of the University of Malawi. However, in the investigated areas only three nonlicensed vendors were found who sold the sampled types of medicines. In total, the samples for this study were collected from 10 public health centers, four public district hospitals or district health offices, one public central hospital, eight CHAM facilities, two licensed pharmacies, three licensed drug stores, and three nonlicensed street vendors.

### Sample collection and transportation.

Permission for the collection of samples in the districts was obtained from the respective District Health Officer. It was not announced beforehand which facilities were visited in a certain district, and on which dates. In public and CHAM facilities as well as in private pharmacies/drug stores, the investigators identified themselves and asked for permission to collect medicine samples from the dispensary area. In order not to cause drug stock-outs by the sample collection, a replacement for the sampled medicines was offered using prepacked medicines carried by the investigators.^28^ In illegal outlets, samples were collected using mystery shoppers, that is, by Malawi nationals from the respective region who acted as customers or patients, not identifying themselves as investigators. The mystery shoppers stated that they had been asked by friends in their village to buy these medicines for them.

Samples of 150 tablets/capsules or 50 vials were collected if available, otherwise smaller numbers. Each sample was recorded separately on a sample collection form. Samples were collected in their original primary and secondary packaging if possible. If the health facility distributed the dosage forms from bulk containers (e.g., polyvinyl chloride [PVC] bottles), samples were collected from these bulk containers and transferred into screw-cap PVC bottles carried by the investigators. Cotton wool was placed inside these containers as protection from mechanical stress. Photos of the original container and its label were taken at the collection site. All packages belonging to the respective sample were immediately labeled with a unique sample code, using preprinted adhesive labels. Samples were transported within 48 hours to the Pharmacy Department, College of Medicine, Blantyre, and stored below 25°C until analysis. The samples selected for subsequent pharmacopeial analysis were forwarded by courier service at ambient temperature to the WHO-prequalified quality control laboratory in Nairobi, Kenya.

### Testing laboratories.

Visual and physical examination and rapid screening by thin-layer chromatography (TLC) and disintegration testing was carried out at the Pharmacy Department, College of Medicine, University of Malawi. Testing according to pharmacopeial monographs was carried out at the WHO-prequalified quality control laboratory of the Mission for Essential Drugs and Supplies (MEDS) in Nairobi, Kenya.^29^

### Quality tests performed and test methods and specifications used.

Visual inspection, disintegration testing, and TLC were carried out according to the manual of the GPHF-Minilab (Global Pharma Health Fund e.V., Gießen, Germany).^30^ In short, the external packaging, primary packaging and (if available) package leaflets were inspected, including batch number and expiry dates. The tablets were visually inspected, especially for undamaged, unaltered surfaces, and color uniformity. Primary and secondary packaging, package leaflet, and the individual dosage forms were documented using a digital camera.

For disintegration testing of instant-release oral dosage forms, six tablets or capsules were kept in water at 37°C under occasional shaking or stirring, and disintegration within 30 minutes was observed. In case not all of the tablets disintegrated, the test was repeated three times. TLC was done according to the procedure given by the manual of the GPHF-Minilab for the respective medicine.^30^ From each sample, three tablets were analyzed individually. Typically, tablets were crushed to a fine powder and extracted with a defined volume of the appropriate solvent by vigorous shaking for 3 minutes. After sedimentation of undissolved residues, an aliquot of the supernatant was removed and appropriately diluted with the respective solvent. Using a microcapillary, 2 μL of this solution were applied to a TLC plate (Merck silica gel 60 F254, 0.2-mm thickness, 5 × 10 cm). Authentic standard solutions of the active pharmaceutical ingredient (API), prepared from standards supplied with the GPHF-Minilab, were applied as comparison. The plate was developed in the appropriate solvent system. After drying off the residual solvent, the APIs were visualized as described in the GPHF-Minilab manual for the respective compound. In most cases, the active ingredients were visualized first under ultraviolet light (254 nm), and subsequently by iodine vapor. The TLC results were documented using a digital camera (Canon Power Shot SX600 HS, Canon Germany GmbH, Giessen, Germany).

All abovementioned tests were carried out for all 155 eligible samples, and the results were recorded on a separate laboratory analysis form for each sample. Fifty-six samples were selected for analysis according to pharmacopeial monographs. These included four samples that had failed prescreening tests (TLC or disintegration time), five further samples which had shown minor defects of the dosage forms in visual inspection, as well as one sample sold by a nonlicensed street vendor with no indication of product name, manufacturer, and expiry date. In addition to these 10 suspicious samples, 46 samples were chosen at random, sampling from each type of medicine. For budgetary reasons, the analysis of samples from this study was integrated into the workflow of the routine analyses of medicines procured by MEDS, a large drug supply organization. The fraction of samples of each type of medicine, which was randomly selected for pharmacopeial testing therefore varied (see Table 1) according to the time slots available in the routine workflow. Not less than two samples of each medicine were randomly selected for pharmacopeial testing.

The samples were analyzed according to the specifications of the pharmacopeia indicated by the manufacturer on the product label. Depending on the respective monograph, these tests included identity; assay for APIs declared on the label; for solid dosage forms, dissolution of the APIs and uniformity of dosage units (by mass as well as by content); for liquid dosage forms, pH value, and volume in containers.

If no pharmacopeia was indicated by the manufacturer, the following pharmacopeial monographs were applied:

- USP38-NF33 for amodiaquine tablets; amoxicillin capsules; amoxicillin tablets; amoxicillin/clavulanic acid tablets (dissolution testing according to USP36-NF31); cefuroxime axetil tablets; ciprofloxacin tablets; phenoxymethylpenicillin tablets; and sulfadoxine/pyrimethamine tablets.
- BP 2015 for amoxicillin capsules; amoxicillin dispersible tablets; chloramphenicol capsules; quinine hydrochloride injection; and quinine sulfate tablets.
- International Pharmacopeia for artemether/lumefantrine tablets and artesunate tablets.
- MEDS in-house methods for dihydroartemisinin/piperaquine tablets (identity, assay, and dissolution).

### Definition of compliance of samples with standards.

In visual and physical examination, samples were considered as noncompliant if the information on the primary and secondary packaging was inconsistent or incorrect. If the dosage forms showed defects like discolorations or cracks, this was noted in the laboratory analysis form but the sample was not classified as noncompliant unless further tests failed.

In disintegration testing according the GPHF-Minilab manual,^30^ samples were considered as noncompliant if, in three tests with six dosage units each, more than two out of 18 dosage units did not disintegrate in 30 minutes. The present study only comprised solid oral dosage forms, which are expected to disintegrate in 30 minutes, no slow-release or enteric-coated tablets.

In TLC according to the GPHF-Minilab manual, samples were considered noncompliant if the retention factor value (Rf) of the APIs did not match that of the authentic standards, and/or if the intensity of the spot was less than that of a reference containing 80% of the stated amount of the API. In TLC, the Rf is the ratio of the distance traveled by the API divided by the total distance traveled by the mobile phase. If a sample failed, TLC analysis was repeated twice before concluding noncompliance.

In testing according to pharmacopeial monographs, samples were considered noncompliant if they failed the specifications of the respective monograph. Dihydroartemisinin/piperaquine tablets, which were investigated according to MEDS in-house methods, were considered noncompliant if the content of either or both APIs deviated by more than 5% from the stated amount, or if less than 70% of either or both APIs dissolved in dissolution medium in 60 minutes.

### Ethical approval.

The study was approved by the College of Medicine Research and Ethics Committee under number P.05/14/1571, as well as by the national drug regulatory agency, that is, the Pharmacy, Medicines and Poisons Board of Malawi (PMPB). PMPB requested that product names, manufacturers, and batch numbers of samples failing pharmacopeial specifications would be communicated to PMPB for appropriate action, but would not be revealed to the public by the investigators.

## Results

### Overview of the samples collected.

We collected samples of the six antimalarial and six antibiotic medicines shown in Table 1. The Malawi Essential Medicines Lists of 2009 and 2015 specify the level of health institution at which each medicine is normally permitted for use: only at central hospitals; or at both central and district hospitals; or on all levels of health-care facilities including health centers. The 12 sampled medicines contain examples from each of these three levels of use, as well as medicines not contained in the Malawi Essential Medicines List (Table 1).

In Malawi, the first-line treatment of malaria is artemether/lumefantrine tablets.^31^ This medicine is available free of charge not only in public but also in CHAM health facilities, provided to the Malawi health-care system through donor-funded programs. Sulfadoxine/pyrimethamine tablets are intended for the intermittent presumptive treatment of malaria in pregnancy.^31^ Quinine injections are used for the treatment of severe malaria in adults.^31^ Artesunate/amodiaquine tablets are the second-line treatment of malaria in Malawi, used in cases when the first-line treatment is ineffective.^31^ Quinine sulfate tablets are used for the treatment of uncomplicated malaria in the first trimester of pregnancy, combined with clindamycin.^31^ Dihydroartemisinin/piperaquine tablets are included into the WHO guidelines for the treatment of malaria,^32^ but not into the Malawi Essential Medicines List. In our study, it was only found in private facilities.

The antibiotic phenoxymethylpenicillin was included the Malawi Essential Medicines List of 2009.^33^ After the present study had been initiated, the Malawi government published a revised version of its essential medicines list,^31^ eliminating the use of phenoxymethylpenicillin in the public health-care system and replacing it with the broad-spectrum antibiotic amoxicillin.

Amoxicillin/clavulanic acid is used for the treatment of infections with bacteria resistant to conventional penicillins due to production of β-lactamases.^31^,^34^ Ciprofloxacin is a widely used (and overused) gyrase inhibitor.^31^,^34^ Chloramphenicol is used, for example, in the treatment of severe *Haemophilus influenzae b* infections and of typhoid fever.^31^ However, the Malawi Essential Medicines Lists of 2015 does not include oral chloramphenicol any longer. Nevertheless, the drug was available in many of the public and CHAM facilities. Cefuroxime tablets are the most frequently prescribed cephalosporin in several industrialized countries, including Germany,^35^ but it is not included in the WHO Essential Medicines List and neither in the Malawi Essential Medicines Lists of 2009 or 2015. In our study, it was only found in the central hospital and in private drug outlets.

At each collection site, one sample of each medicine listed in Table 1 was collected if available. As expected, medicines which according to the Malawi Essential Medicine List should be available on all levels of the health-care system could be collected in many facilities, whereas medicines restricted to central hospitals or not included into the Malawi Essential Medicine List were only available in few facilities (Table 1).

A total of 158 samples were collected, but three had to be excluded from analysis: one expired on the month of collection, and for two samples the number of units collected was too small for analysis. All other 155 samples were included in the analysis.

### Sites of sample collection.

Medicines were collected in four districts of southern Malawi from 10 randomly selected public health centers, from the public hospitals of each district, and from CHAM facilities, licensed private pharmacies and drug stores, and nonlicensed street vendors, which were located near these public health facilities (see the Methods section). As shown in Table 1, the collection sites comprised 15 public and 16 CHAM or private facilities. Storage and transportation conditions of the samples are described in the Methods section.

### Countries of origin, manufacturers, and batches.

As shown in Table 2, the medicines collected came from 33 different manufacturers, located in 12 different countries. According to the information on the labels, approximately 50% of the samples came from India, and another 25% from Kenya and China. The 12 medicine samples from the United States were Coartem™ samples, an artemether/lumefantrine combination from Novartis (Suffern, NY). Seven samples (5%) were produced in Malawi, by two different manufacturers.

Batch numbers were available for 150 samples, representing 123 different batches. In 17 cases, two samples belonged to the same batch, and in five cases, three samples belonged to the same batch. Samples belonging to the same batch invariably showed identical results in prescreening analysis. In three instances, two or more samples belonging to the same batch were analyzed according to pharmacopeial monographs. The determined content of the active ingredient varied between the samples of the same batch by ±2.1%, ±0.5%, and ±0.2%, respectively, and the dissolution of the active ingredient varied by ±1.9%, ±5.3%, and ±3.5%, respectively. This shows consistency of the analytical results, even though the samples may have been exposed to different storage conditions before collection.

### Registration and WHO prequalification status of sampled products.

The registration status of the collected medicines was checked using the records of the national drug regulatory agency, that is, the PMPB. As shown in Tables 2 and 3, 111 samples (representing 50 different medicines) were registered, whereas 43 samples (representing 13 different medicines) were not.

WHO has established the Prequalification of Medicines Program to assist in the selection of good-quality medicines for international procurement.^36^ A list of WHO-prequalified medicines is available on the WHO website.^37^ Out of the 12 types of medicines included into this study, three were included into the WHO Prequalification Program at the time of sample collection (artemether/lumefantrine tablets, artesunate/amodiaquine tablets, and ciprofloxacin tablets). Twenty-eight samples (from four manufacturers) represented WHO-prequalified medicines, and none of them failed testing (Table 3).

### Generic and brand name medicines and types of primary packaging.

As shown in Table 3, 50% of the samples were generic medicines sold under their international nonproprietary names, and 42% represented so-called branded generics, that is, generic medicines sold under brand names given by the manufacturers. The only originator brand encountered in this study was the abovementioned Coartem.

The primary packaging of most medicines was blister packs (46%) and PVC bottles (43%) (Table 3). Quinine injections were the only injectable medicine included in this study, with glass ampoules as primary packaging. Two medicines were packed in aluminum strip packs, and two further medicines in paper strip packs.

### Compliance with specifications: overall results.

All 155 samples were analyzed by rapid laboratory screening methods (TLC and disintegration testing) as well as by visual and physical examination. Four samples failed the laboratory prescreening, and five further samples showed defects in the dosage forms in visual examination.

Fifty-six samples were tested according to pharmacopeial monographs. Of these, seven failed pharmacopeial analysis, with one sample classified as falsified, three samples classified as substandard with extreme deviations from pharmacopeial standards, and three samples as substandard with nonextreme deviations (Table 4).

### Compliance with specifications: visual and physical examination.

One sample, labeled as a Malawian branded generic medicine of sulfadoxine/pyrimethamine 500 mg/25 mg, failed visual inspection (Table 4, sample A). It contained two types of paper strips with similar appearance but different stamps. One type was correctly stamped with batch number and expiry date. The other type was stamped with two dates, “April 27, 2010” and “December 20, 2015”; the irregular interval between these dates suggested that they may not represent correct dates of manufacture and expiry. This latter type of paper strips was found to contain two different types of tablets. Both were clearly different from the genuine medicine of the Malawian manufacturer, as visible from the tablet imprints. This strongly suggested that they represented falsified medicines. TLC and pharmacopeial analysis showed that they did not contain sulfadoxine/pyrimethamine. One type was identified as paracetamol 500 mg tablets, the other type co-trimoxazole 480 mg tablets. Because of the absence of the declared active ingredients, and the presence of other active ingredients, these falsified medicines represented a serious public health risk. The national drug regulatory agency and the WHO Medical Product Alert System were informed, and a preliminary report describing details of this falsified medicine and its identification was published.^24^

Visual and physical examination identified six further samples, which showed some visible defect of the dosage units (in all cases tablets). One of them (Table 4, sample D) also failed in disintegration testing in the prescreening, and subsequently in dissolution testing. Only one of the other five samples with visible defects failed pharmacopeial analysis (Table 4, sample B), whereas four complied with pharmacopeial specifications.

### Compliance with specifications: rapid prescreening tests.

Rapid prescreening using the GPHF-Minilab comprised TLC and disintegration testing.^30^ In addition to the falsified sulfadoxine/pyrimethamine sample mentioned earlier, two samples of phenoxymethylpenicillin 250 mg tablets were considered to fail TLC analysis: the spots of the API observed in TLC (on visualization with iodine staining) were considered to show less intensity than those of a standard containing 80% of the declared amount of phenoxymethylpenicillin. Pharmacopeial analysis, however, showed that they contained 94.2% and 94.5% of the declared amount of the API, respectively, and therefore complied with the specification of USP38-NF33.

One sample of (Table 4, sample D) failed disintegration testing. As mentioned earlier, this sample had also shown visible defects of the tablets. Pharmacopeial analysis confirmed it to be substandard with an extreme deviation of the dissolution of the API (Table 4).

### Compliance with specifications: analysis according to pharmacopeial monographs.

Ten samples which in prescreening failed or appeared suspicious (see the Methods section: three TLC failures, one disintegration failure, five samples showing defects in dosage form, and one unlabeled sample from an unlicensed vendor) as well as 46 randomly selected samples (see the Methods section) were subjected to analysis according to pharmacopeial monographs. Of these 56 samples, seven failed pharmacopeial analysis, and details of these samples are shown in Table 4. Of these, one sample (Table 4, sample A) was considered as falsified (as mentioned earlier), due to absence of both stated APIs and presence of other APIs. Three samples (Table 4, samples B, C, and D) were considered as substandard with extreme deviations from the pharmacopeial specifications following the criteria defined in a study by WHO on medicine quality in Africa,^20^ that is, showing more than 20% deviation from the stated content of the API, or a dissolution of the active ingredient more than 25% below the limit defined in the relevant pharmacopeia. Three further samples (Table 4, samples E, F, and G) were classified as substandard, but with nonextreme deviations. An example of such nonextreme deviations are two samples of chloramphenicol tablets containing 109% and 110% of the stated amount of the API (Table 4, samples F and G) and thereby falling outside of the limits of 95–105% stated in the British Pharmacopeia 2015 (Table 4). The national drug regulatory agency and the WHO Medical Product Alert System were informed about the failed samples, their manufacturers and their batch numbers.

## Discussion

### Testing methods and data quality.

In the present study, rapid screening tests (disintegration testing and TLC) were carried out using the GPHF-Minilab.^30^,^38^ As discussed below, TLC is a sensitive method to detect falsified medicines. Therefore, the overall prevalence of falsified medicines observed in our study (one out of 155 samples) is 0.6% (95% confidence interval [CI] = 0.01–3.47%).^39^

In contrast, Minilab testing is of limited sensitivity in the detection of substandard medicines.^20^ Therefore, the prevalence of substandard medicines needs to be based on the number of samples evaluated according to pharmacopeial monographs. Six of these samples (11%) were found to be substandard, in addition to the one falsified sample.

Pharmacopeial analysis of 56 samples was carried out in a WHO-prequalified quality control laboratory,^29^ using state-of-the-art methods. Sample collection, prescreening, and documentation were carried out under close supervision of experienced research pharmacists, ensuring adequate data quality.

### Interpretation of results and recommendations.

The most interesting result of the present study is that no falsified medicines and only a single substandard medicine were identified in public and CHAM health facilities. This is in contrast to the image purported in local news reports^16^ and to the opinion held by many health professionals in Malawi. On first glance, it also appears to be in contrast with reports in credible scientific journals on the high prevalence of substandard and falsified medicines in Africa.^4^ Yet, a careful analysis of the scientific literature, considering the difference between falsified and substandard medicines, as well as the marked differences in the occurrence of poor-quality medicines between different regions of Africa and between drug outlets of the formal and the informal sector, shows that the results of the present study are in agreement with the current scientific knowledge.

An investigation of 935 medicine samples from six countries of sub-Saharan Africa carried out by the WHO QAMSA (Quality of Selected Antimalarial Medicines Circulating in Six Countries of Sub-Saharan Africa) study^20^ found only two samples (0.2%) in which a stated active ingredient was missing entirely. A meta-analysis of studies carried out by the Promoting the Quality of Medicines Program of the U.S. Pharmacopeial Convention (USP-PQM) in the period 2003–2013 reported on a total of 3,371 medicine samples collected in different African countries.^12^ Only 11 (0.3%) of the samples were falsified. The ACT Consortium Drug Quality Program (ACTcDQP) investigated, in several studies, the quality of 10,079 medicine samples from six countries, including five African countries.^13^ Ninety-eight samples (0.97%) were found to be falsified. All these results are similar to those of our study.

In addition to the one falsified medicine, six samples (11%) in the present study were found to be substandard. The meta-analysis of USP-PQM reported 350 out of 3,371 samples from Africa (10.4%) to be substandard,^12^ whereas ACTcDQP found 779 out of 10,079 medicine samples (7.7%) to be substandard.^13^ In comparison to the USP-PQM studies, the ACTcDQP studies underestimate the prevalence of substandard medicines, as they did not investigate dissolution of the APIs and used wider tolerance limits for the quantity of the APIs than the U.S. Pharmacopeia. The WHO QAMSA study found that in east African countries (Ethiopia, Kenya, and Tanzania) seven out of 127 samples (5.5%) were out of specifications, whereas in west African countries (Nigeria, Ghana, and Cameroon) the prevalence was 69 out of 140 samples (49%). This highlights the strong regional differences in the occurrence of poor-quality medicines.

In the present study, the single falsified medicine sample, which was identified was collected in the informal sector, that is, from a nonlicensed street vendor. In addition, five substandard medicines were found in different parts of the private sector (two licensed pharmacies, two licensed drug stores, and one nonlicensed street vendor). Therefore, the prevalence of out-of-specification medicines in the (licensed and nonlicensed) private facilities was 6/21 (29%), in contrast to 1/35 (3%) in the public and faith-based facilities (Table 3). This difference is striking. Despite the small sample size, it is statistically significant in the “N-1” χ^2^ test (*P* = 0.005), although the 95% CI for the difference is large (*D* = 26%; 95% CI = 4.7–49.8%).^40^

Few previous studies have distinguished between medicine quality in public, faith-based, and licensed private facilities, but several studies have proven a higher prevalence of poor-quality medicines in the informal sector as compared with the formal sector.^8^,^20^,^41^,^42^ Therefore, when data on the prevalence of poor-quality medicines are compared between different studies, it is important to consider the types of collection sites, especially the proportion of samples collected in the informal sector. In the WHO QAMSA study,^20^ the USP-PQM studies^12^ and the ACTcDQP studies,^13^,^43^ the large majority of the samples came from the formal sector, similar to our present investigation. Studies of the informal sector would produce a very different picture,^8^,^20^,^41^,^42^ and this is also suggested by the few samples from the informal sector included into the present study (Table 3).

While the present study was in progress, a survey of the quality of antimalarial medicines in Malawi was published by Chikowe and others.^44^ The 112 samples of that survey had been collected 3 years before our study. They were obtained exclusively from private (licensed or nonlicensed) facilities, not from public or CHAM facilities. The samples were analyzed for the content of the APIs using nonpharmacopeial high-performance liquid chromatography (HPLC) methods. Dissolution of the APIs was not tested. None of the 112 samples was found to be falsified, but an unusually high proportion (88.4%) was reported to be substandard due to insufficient or excessive amounts of the APIs. Because of the different time of collection, the results are not directly comparable to our study. Yet, in our study only three out of 21 samples (14%) from private licensed or nonlicensed facilities showed an incorrect amount of the API (Tables 3 and 4). Surprisingly, and in contrast to our study, Chikowe and others^44^ reported wide variations in API content within medicines of the same batch. HPLC chromatograms depicted in the publication^44^ show very irregular peak shapes, suggesting that there may have been problems with the analytical methods.

Our finding of a low prevalence of substandard and falsified medicines in public and CHAM health facilities may help to restore the trust of both the population and the health workers in the medicines provided in these facilities. This is important, as distrust may lead to an underutilization of the facilities by the population, and also may undermine adherence to treatment guidelines by health workers and patients. Both effects are likely to result in increased morbidity and mortality.

Despite the encouraging finding regarding medicine quality in public and CHAM facilities, the present study confirmed that poor-quality medicines are in circulation in Malawi, especially in the private for-profit sector. This includes medicines with extreme deviations from pharmacopeial standards and falsified medicines. Continued efforts are required to identify and combat the occurrence of such medicines. For example, attention needs to be given to the manufacturing standards of local manufacturers. The WHO QAMSA study showed that in several African countries the failure rates of medicines from domestic producers were higher than those from imported products.^20^ Also in the present study, two out of five Malawian medicines investigated according to pharmacopeial methods were found to be substandard (Table 2). These samples represented two different medicines from the same manufacturer (Table 4, samples D and E). The national drug regulatory agency was alerted to this finding.

It is frequently suspected that low-quality medicines found in Africa derive especially from India. Our study presents two facts on this question. On the one hand, 22 out of 25 fully tested samples of medicines from India were found to comply with the pharmacopeial specifications. On the other hand, three samples stated to come from India were found to be substandard, two of them even with extreme deviations (Table 4). Notably, two of the substandard medicines came from the same manufacturer, and again the national drug regulatory agency was alerted to this problem. Three substandard medicines from India are three too many, and this calls for action by the drug regulatory authorities both in India and in Malawi.

None of the WHO-prequalified medicines^36^ investigated in this study failed the analysis. This is encouraging, and purchasing WHO-prequalified medicines is undoubtedly useful to ensure medicine quality. However, the sample size of the present study is too small to provide conclusive evidence for a higher quality of WHO-prequalified medicines. Such evidence has been provided in a previous study.^20^

Out of 63 different medicines collected in this study, 13 (representing 43 samples) were not registered in Malawi (Tables 2 and 3). Similar findings have been reported previously both from Malawi^44^ and from other African countries.^20^ Better registration coverage is certainly desirable, but the present study suggests that this in itself will not influence the quality of medicines in Malawi, since all identified poor-quality medicines were registered (Table 3).

### Predictive value of prescreening tests.

The present investigation followed the 3-level approach used by the USP-PQM and the WHO QAMSA studies,^12^,^20^,^21^ using consecutively:

- visual and physical examination of the medicines and their packaging;
- rapid prescreening with the GPHF-Minilab;
- and testing according to pharmacopeial monographs, including dissolution testing.

The usefulness and limitations of the prescreening methods have been evaluated in detail by the WHO QAMSA study,^20^ and our results are in accordance with that evaluation. Visual examination allows the immediate identification of some falsified/counterfeit medicines, as also exemplified in the present study.^24^ Visual and physical examination can furthermore detect failures in the appearance of the dosage forms (e.g., erosions and discolorations). However, outcomes of these assessments correlate poorly with outcomes of pharmacopeial testing.^20^ In the present study, out of seven samples showing failures in the appearance of the dosage forms, two failed pharmacopeial testing (Table 4, samples B and D), five did not.

Disintegration testing is a useful prescreening method. Disintegration of a solid oral dosage form is a necessary but not a sufficient condition for dissolution of the API, which in turn is a prerequisite for bioavailability. The Minilab disintegration test therefore is a quite specific, albeit insensitive, pretest for dissolution. In the WHO QAMSA study,^20^ five samples were found to fail in the disintegration test, and indeed four of them subsequently failed dissolution testing. On the other hand, 31 further samples failed dissolution, despite passing the disintegration test. In the present study, a single sample failed disintegration, and subsequently indeed showed an extreme deviation in dissolution testing (Table 4, sample D). Three further samples failed dissolution, despite passing the disintegration test (Table 4, samples B, C, and E).

TLC is a quite specific and sensitive test for falsified medicines, defined as medicines which contain either no or incorrect active ingredients.^12^,^13^ In the WHO QAMSA study, two falsified medicines were identified, both lacking one of two stated APIs.^20^ Both samples were identified already in TLC analysis.^20^ In the present study, the power of TLC was strikingly exemplified in the identification of a falsified sulfadoxine/pyrimethamine sample.^24^

TLC is primarily a qualitative method, with limited sensitivity and specificity in the detection of incorrect quantities of APIs, unless the deviation is very high. In the WHO QAMSA study, 41 samples were found to contain incorrect amounts of APIs, 17 of which had already been detected in TLC analysis. On the other hand, seven further samples which were considered to fail the quantitative evaluation of the TLC analysis were subsequently found to contain the correct amount of APIs. In the present study, three samples contained incorrect amounts of APIs (Table 4, samples B, F, and G), but none of those were detected in TLC analysis. In contrast, two samples which were considered to fail the quantitative evaluation of the TLC analysis were later found to contain the correct amount of APIs. The experience of the investigator has a strong influence on the sensitivity and specificity of the quantitative evaluation of the TLC results. Notably, a method to improve the quantitative evaluation of Minilab TLC results using photography and an imaging software has recently been reported.^45^

### Costs of Minilab and pharmacopeial analysis.

For the present study, a GPHF-Minilab including TLC solvents and reference standards was purchased for US$6,400. Airfreight to Malawi costed another US$970. The materials contained were more than sufficient to run the analysis of the 155 samples of this investigation.

At the same time, we also requested a quotation from a WHO-prequalified laboratory in the Republic of South Africa for a pharmacopeial analysis of the 12 medicines investigated in this study. Based on this quotation and including a discount offered, the cost for an analysis of the 155 samples listed in Table 1 would result as US$245,000 (Supplemental Table 1). The quote included all pharmacopeial tests carried out in the present study, but only up to Stage 1, that is, excluding the repetitions required when a sample fails in the first test. Testing for related substances was not included in the quotation, and neither was it carried out in the present study. The prices quoted were similar to those charged by European laboratories. Cheaper offers for medicine quality analysis may be obtained from some WHO-prequalified medicine control laboratories^29^ in India (C. Haefele-Abah, personal communication).

US$245,000 may be affordable for research projects in industrialized countries, but not for low-income countries. This may become even more obvious from a comparison to the purchasing price of the medicines in question. Using the Management Sciences for Health/WHO reference price for international procurement,^46^ the 155 medicine samples listed in Table 1 (150 tablets/capsules or 50 vials per sample) can be purchased for US$1,840 (Supplemental Table 1). Following the Malawi Standard Treatment Guidelines,^31^ this quantity of the 12 different medicines is sufficient for 2,220 courses of treatment of the most common diseases treated with these drugs. The quoted cost for the pharmacopeial analysis of the 155 medicine samples (US$245,000) would therefore be equivalent to the procurement costs for 295,000 courses of treatment with these medicines (Supplemental Table 1). A country like Malawi cannot afford to leave tens or hundreds of thousands of sick patients untreated to analyze 155 medicine samples.

Therefore, there is an urgent need for low-cost screening technologies to help in the detection of poor-quality medicines in low- and middle-income countries. One of the very few commercially available technologies is the GPHF-Minilab,^30^,^38^ recently described as “remaining a key component of drug quality surveillance systems” in developing countries.^13^

As correctly pointed out in the report of the WHO QAMSA study,^20^ use of the Minilab alone is not sensitive enough to reliably detect substandard medicines. However, use of pharmacopeial methods alone is too expensive for routine surveillance and for larger surveys in low- and middle-income countries. The solution of this dilemma most likely lies in a compromise: large sample numbers can be screened with technologies like the Minilab, identifying most of the falsified medicines as well as a certain proportion of the substandard medicines. All samples failing in the prescreening, and a random selection of the samples passing the prescreening, are then analyzed by pharmacopeial methods, to confirm the Minilab results and to reliably detect the occurrence of substandard medicines in this smaller subsample.

In such a two-level study design, inclusion of all medicines which failed prescreening into the pharmacopeial analysis introduces a bias toward poor-quality medicines. For example, in the present study, overall seven out of 56 samples (12.5%) failed pharmacopeial analysis. Of the 46 randomly selected samples, only four (8.7%) failed pharmacopeial analysis. The true prevalence of out-of-specification medicines would be in between these two values (±statistical error).

### Limitations of this study.

The present study was of limited size. It focused on medicines from public and faith-based health facilities, with very few samples collected from the informal market. This is similar to the cited WHO QAMSA study which collected a mean number of 156 samples from each country, selecting 45 for pharmacopeial analysis; 3.5% of these samples were from the informal market (in the three east African countries).^20^ Because of the similar size and methodology, the results of the present study can be well compared with the results obtained for six other African countries in the WHO QAMSA study.

We used an overt approach in the collection of medicines from public and CHAM facilities, as well as from private pharmacies and drug stores. This may lead to a sampling bias in favor of good-quality medicines. However, previous studies have shown identical results for overt and mystery shopping.^43^,^47^

Although all samples collected in this study were prescreened using the GPHF-Minilab, only one-third could be analyzed by pharmacopeial methods due to budget constraints. For the same reason, testing for related substances was not carried out in this study. Tests for related substances are included in the pharmacopeial monographs primarily to control degradation impurities of the APIs, and to limit impurities arising during synthesis. Testing for related substances is expensive and rarely included in medicine quality studies in developing countries. Even in the WHO QAMSA study, is was only carried out for two of the five types of medicines.^20^

## Supplementary Material

Supplemental Table.

## Figures and Tables

**Table 1:** Overview of the number of analyzed medicine samples collected from different types of health facilities

Availability according to MEML*		Medicine	Public health facilities (*N* = 15)	CHAM facilities (*N* = 8)	Licensed pharmacies and drug stores (*N* = 5)	Nonlicensed medicine vendors (*N* = 3)	Total no. of samples	Samples tested according to pharmacopeial monographs
2009	2015							
H	H	Artemether/lumefantrine 20 mg/120 mg tbl	14	8	4	0	26	6
H	H	Sulfadoxine/pyrimethamine 500 mg/25 mg tbl	14	8	4	2	28	11
H	H	Quinine hydrochloride inj. 300 mg/mL, 2 mL vial	8	3	1	0	12	2
H	–	Phenoxymethylpenicillin 250 mg tbl	0	5	2	2	9	3
D	H	Amoxicillin 250 mg cps/tbl	9	5	3	2	19	6
D	D	Artesunate/amodiaquine 100 mg/270 mg tbl	5	0	1	0	6	2
D	D	Quinine sulfate 300 mg tbl	3	6	3	0	12	10
C	D	Ciprofloxacin 250 mg tbl	9	8	3	1	21	4
C	C	Amoxicillin/clavulanic acid 500/125 mg tbl	0	1	2	0	3	2
N	–	Chloramphenicol 250 mg cps	4	5	3	0	12	6
–	–	Dihydroartemisinin/piperaquine 40 mg/320 mg tbl	0	0	3	0	3	2
–	–	Cefuroxime (as axetil) 250 mg tbl	1	0	2	1	4	2
			67	49	31	8	155	56

CHAM = Christian Health Association of Malawi; MEML = Malawi Essential Medicines Lists.

* The MEML of 2009 and 2015 specify the level of health institution at which the medicine is normally permitted for use: H = at health center, district hospital, and central hospital levels; D = at district hospital and central hospital levels only; C = at central hospital level only; N = level of use not specified; and – = not included in MEML.

**Table 2:** Origin of the investigated medicines

Stated country of origin	No. of samples	No. of manufacturers	No. of different medicines	No. of unregistered medicines	Samples tested according to pharmacopeial monographs	Samples failing pharmacopeial specifications
India	78	15	31	6	25	3 (12%)
Kenya	22	4	11	3	8	1 (13%)
China	17	4	7	2	4	0 (0%)
United States	12	1	1	0	2	0 (0%)
Malawi	7	2	3	0	5	2 (40%)
Morocco	5	1	1	0	2	0 (0%)
Tanzania	4	1	2	0	4	0 (0%)
Cyprus	3	1	3	0	2	0 (0%)
Austria	2	1	1	1	0	n.a.
Switzerland	1	1	1	0	0	n.a.
United Arab Emirates	1	1	1	1	1	0 (0%)
United Kingdom	1	1	1	0	1	0 (0%)
Unknown (lack of label)*	1	n.a.	n.a.	n.a.	1	0
Unknown (falsified medicine)†	1	n.a.	n.a.	n.a.	1	1
Total	155	33	63	13	56	7

n.a. = not applicable.

* Sold by a nonlicensed street vendor, with no indication of product name, manufacturer, and expiry date.

† Labeled as a medicine from a Malawian manufacturer.^24^

**Table 3:** Differentiation of medicine samples by collection site, registration and WHO prequalification status, generic versus brand name medicines, and primary packaging

	Total no. of samples	Samples tested according to pharmacopeial monographs	Samples failing pharmacopeial specifications	
All samples	155	56	7 (13%)	
Type of collection site				
Public health facility	67	24	1 (4%)	3%
CHAM facility	49	11	0 (0%)	
Licensed pharmacy/drug store	31	16	4 (25%)	29%
Nonlicensed vendor	8	5	2 (40%)	
PMPB registration status of sampled medicines				
Registered	111	43	7 (17%)	
Nonregistered	43	12	0 (0%)	
Unknown	1	1	0 (0%)	
WHO prequalification status of sampled medicines				
WHO prequalified	28	7	0 (0%)	
Not WHO prequalified	126	49	7 (14%)	
Unknown	1	1	0 (0%)	
Type of medicine				
Generic medicine	77	25	1 (4%)	
Branded generic medicine	65	28	6 (21%)	
Originator brand medicine	12	2	0 (0%)	
Unknown	1	1	0 (0%)	
Type of primary packaging				
Blister packs	71	25	1 (4%)	
PVC bottle	67	25	3 (12%)	
Aluminum strip packs	2	1	1 (100%)	
Paper strip packs	2	2	2 (100%)	
Glass ampoules	12	2	0 (0%)	
Unknown (sold in plastic bag)	1	1	0 (0%)	

CHAM = Christian Health Association of Malawi; PMPB = Pharmacy, Medicines and Poisons Board of Malawi; PVC = polyvinyl chloride; WHO = World Health Organization.

**Table 4:** Medicine samples not complying with pharmacopeial standards

Medicine	Type of collection site	Regulatory status	Stated country of origin	Manufacturer	Primary packaging	Visual and physical examination	Disintegration testing	Thin-layer chromatography	Pharmacopeial analysis (pharmacopeia)	Reason for noncompliance
Falsified medicines										
A) Sulfadoxine/pyrimethamine 500 mg/25 mg tbl, falsely labeled as a Malawian branded generic	Nonlicensed medicine vendor	Not applicable	Malawi	? label claim: “Mf.1”	Paper strip packs	Inconsistent expiry dates and tablet imprints	Compliant	Noncompliant	Noncompliant (USP-38 NF33)	Identity: absence of both stated APIs; presence of other APIs

Substandard medicines with extreme deviations*										
B) Sulfadoxine/pyrimethamine 500 mg/25 mg tbl, branded generic “A”	Nonlicensed medicine vendor	Registered	Malawi	“Mf.1”	Paper strip packs	Chippings on tablet surface	Compliant	Compliant	Noncompliant (USP-38 NF33)	Assay: sulfadoxine 71.8% of stated amount. Dissolution of sulfadoxine: average 53.2% of stated amount. Dissolution of pyrimethamine: average 58.8% of stated amount
C) Sulfadoxine/pyrimethamine 500 mg/25 mg tbl, branded generic “B”	Licensed drug store	Registered	India	“Mf.2”	Aluminum strip packs	Compliant	Compliant	Compliant	Noncompliant (USP-38 NF33)	Dissolution of pyrimethamine: average 41.3% of stated amount
D) Quinine sulfate 300 mg tbl, branded generic “C”	Licensed pharmacy	Registered	India	“Mf.3”	PVC bottle	Abrasions on tablet coating	Noncompliant	Compliant	Noncompliant (BP 2015)	Dissolution: average 49.4% of stated amount

Substandard medicines with nonextreme deviations										
E) Chloramphenicol 250 mg cps, branded generic “D”	Public hospital	Registered	Malawi	“Mf.1”	PVC bottle	Compliant	Compliant	Compliant	Noncompliant (BP 2015)	Dissolution: average 60.4% of stated amount
F) Chloramphenicol 250 mg cps, branded generic “E”	Licensed pharmacy	Registered	India	“Mf.3”	Blister packs	Compliant	Compliant	Compliant	Noncompliant (BP 2015)	Assay: 109.2% of stated amount
G) Chloramphenicol 250 mg cps, generic medicine “F”	Licensed drug store	Registered	Kenya	“Mf.4”	PVC bottle	Compliant	Compliant	Compliant	Noncompliant (BP 2015)	Assay: 110.1% of stated amount

API = active pharmaceutical ingredient, PVC = polyvinyl chloride.

* Showing more than 20% deviation from the stated content of the active pharmaceutical ingredient or a dissolution of the active ingredient more than 25% below the limit defined in the relevant pharmacopeia.
