# Supplementary material for: Low Prevalence of Substandard and Falsified Antimalarial and Antibiotic Medicines in Public and Faith-Based Health Facilities of Southern Malawi
Source: Am J Trop Med Hyg. 2017 May 3;96(5):1124–35. doi: 10.4269/ajtmh.16-1008 (PMC5417205; doi:10.4269/ajtmh.16-1008)
Supplement: Supplementary file 1 [file SD4.pdf]

Supplemental Table 1

Quoted costs of pharmacopeial analysis of all medicine samples collected in this study, compared with purchasing cost of these medicines

Type of medicine	No. of samples collected	Quoted cost of analysis for first sample (US$)*	Quoted cost of analysis for each subsequent sample (US$)*	Cost of analysis for all samples (US$)	Size of each sample (units)	Total units collected	MSH reference price per unit (U.S. cent)†	Purchasing costs of total units collected (US$)	Units required per course of treatment (= COT)‡	Cost per COT	No. of COTs possible with units collected	No. of COTs possible with US$245,051.25
Artemether/lumefantrine 20 mg/120 mg tbl	26	3,960.00	2,970.00	78,210.00	150	3,900	17.03	664.17	24	4.09	163	21,598
Sulfadoxine/pyrimethamine 500 mg/25 mg tbl	28	2,180.00	1,635.00	46,325.00	150	4,200	3.73	156.66	3	0.11	1,400	186,077
Quinine hydrochloride inj. 300 mg/mL, 2 mL vial	12	1,585.00	1,188.75	14,661.25	50	600	24.00	144.00	6	1.44	100	13,291
Phenoxymethylpenicillin 250 mg tbl	9	1,345.00	1,008.75	9,415.00	150	1,350	1.72	23.22	56	0.96	24	3,204
Amoxicillin 250 mg cps/tbl	19	1,075.00	806.25	15,587.50	150	2,850	1.84	52.44	42	0.77	68	9,019
Artesunate/Amodiaquine 100 mg/270 mg tbl	6	2,950.00	2,212.50	14,012.50	150	900	25.00	225.00	6	1.50	150	19,937
Quinine sulfate 300 mg tbl	12	1,830.00	1,372.50	16,927.50	150	1,800	6.38	114.84	42	2.68	43	5,696
Ciprofloxacin 250 mg tbl	21	1,375.00	1,031.25	22,000.00	150	3,150	2.13	67.10	28	0.60	113	14,953
Amoxicillin/clavulanic acid 500/125 mg tbl	3	1,950.00	1,462.50	4,875.00	150	450	20.00	90.00	21	4.20	21	2,848
Chloramphenicol 250 mg cps	12	1,075.00	806.25	9,943.75	150	1,800	2.55	45.90	40	1.02	45	5,981
Dihydroartemisinin/piperaquine 40 mg/320 mg tbl	3	3,450.00	2,587.50	8,625.00	150	450	40.09	180.41	9	3.61	50	6,646
Cefuroxime (as axetil) 250 mg tbl	4	1,375.00	1,031.25	4,468.75	150	600	13.33	79.98	14	1.87	43	5,696
Total	155			245,051.25				1,843.71			2,219	294,946

Excluding the very inexpensive sulfadoxine/pyrimethamine treatment, the number of COTs possible with US$245,051.25 would result as 108,869. Even this smaller number does not change the argument that a country like Malawi cannot afford to leave such a number of patients untreated to analyze 155 medicine samples.

*Quotation from a World Health Organization-prequalified laboratory in South Africa for pharmacopeial analysis of the medicines investigated in this study.

†Median supplier price from International Drug Price Indicator Guide.[42]

‡According to Malawi Standard Treatment Guidelines, 2015.[31]
